# Triumphs of Immunization

**DOI:** 10.1093/infdis/jiab123

**Published:** 2021-09-30

**Authors:** John F Modlin, William Schaffner, Walt Orenstein, Ananda S Bandyopadhyay

**Affiliations:** 1 Departments of Pediatrics and Medicine, Geisel School of Medicine, Hanover, New Hampshire, USA; 2 Department of Health Policy, Vanderbilt University School of Medicine, Nashville, Tennessee, USA; 3 Medicine, Pediatrics, Global Health, and Epidemiology, Emory University School of Medicine, Atlanta, Georgia, USA; 4 Bill & Melinda Gates Foundation, Seattle, Washington, USA

**Keywords:** vaccines, immunization impact, global health

The global coronavirus pandemic we now confront has led to unprecedented mortality, strained our medical systems, disrupted our daily lives, and created economic stress unlike any other event in our lifetime. The origin is a novel virus that has swept across the globe with surprising speed, aided by air travel and a complete absence of population immunity. There can be no clearer reminder of the importance of immunization to modern civilization.

The aim of this supplement is to underscore the unparalleled human benefits that have accrued from vaccine discovery and use before the pandemic. In the past 100 years, vaccines that prevent or modify >30 human infectious diseases have been licensed by the United States (US) Food and Drug Administration and other regulatory authorities. The pace of discovery has accelerated in the 21st century with not just numerous new microbial pathogen targets, but also human cancers, allergies, and autoimmune diseases including type 1 diabetes [1, 2]. Advances in molecular biology and manufacturing technologies now promise shorter vaccine development timelines and more cost-efficient production processes. And with increasing innovation and support from philanthropic foundations and nongovernmental organizations for vaccine purchase and delivery, public immunization programs now rank among the most effective and cost-efficient services available.

Vaccines protect in 2 ways. First, by stimulating an active immune response, vaccinated individuals are protected from disease. Second, induction of a critical level of population (or “herd”) immunity mitigates disease burden by interrupting chains of human-to-human transmission so susceptible individuals within the population are indirectly protected because they are not exposed to the vaccine-preventable pathogen. Persons who are indirectly protected include unvaccinated persons including those for whom vaccination is not routinely recommended, those with contraindications to vaccination, and those who fail to receive recommended vaccines, as well as vaccinated persons who fail to mount a protective immune response following vaccination. A striking example is the impact of vaccination of infants and young children <2 years of age with pneumococcal conjugate vaccine in indirectly preventing serious pneumococcal disease in the much larger cohort of adults >65 years of age [3]. Substantial herd immunity benefits also accrue from high coverage levels with other routinely administered vaccines including pertussis, measles, mumps, rubella, varicella, rotavirus, *Hemophilus influenzae* type b, hepatitis A, and human papillomavirus (HPV) vaccines.

The manuscripts included in this supplement herald immunization successes in both the developed and the developing world. In the US, a public-private sector partnership has evolved that includes vaccine manufacturers, community-based medical providers, insurers, state and local health departments, and multiple US government agencies. Through a patchwork of programs, federal legislation supports vaccine purchase and distribution, immunization infrastructure, tort relief for vaccine injury, and insurance coverage mandates. These programs now assure access, regardless of insurance status or ability to pay, to vaccination against 17 diseases for which the Advisory Committee on Immunization Practices (ACIP) has issued universal recommendations across the age spectrum.

The impact is truly unprecedented. Smallpox was eradicated globally 4 decades ago (see Breman in this supplement), 3 diseases (polio, measles, and congenital rubella syndrome) have been effectively eliminated from many developed countries, and morbidity from 6 other diseases has been reduced by >97% in the US [4]. Approximately 95% of children are now protected against diphtheria, tetanus, pertussis, measles, mumps, rubella, and varicella by age 6 years in most US jurisdictions with few racial or socioeconomic disparities, in part due to successful administration of school immunization requirements [5]. Estimates project that vaccines recommended for children in the US and territories will prevent 21 million hospitalizations and avert 730 000 deaths in the 10-year cohort of children born between 1994 and 2013, while saving US$295 billion in direct costs and US$1.38 trillion in societal costs (both net cost of vaccination) [6]. For adolescents and young adults, HPV and meningococcal conjugate vaccine (MenACWY) coverage rates continue to increase in the US, reaching 54% for a completed series of each of these vaccines at age 17 years, respectively, in 2019 [7]. Modeling projects that ≥1-dose HPV coverage will increase to approximately 80% for both males and females by 2022 when the 2012 birth cohort reaches 10 years of age. Since extension of protection to unvaccinated adolescents and young adults can be expected when HPV vaccine coverage rises above 50%, dramatic declines in cervical and other HPV-related cancers can be expected in the next few decades [8–10].

In contrast, improving immunization coverage in adults continues to be a challenge. In the US, about 60%–65% of adults report up-to-date immunization with tetanus toxoid, and a similar proportion of persons aged ≥65 years are vaccinated against pneumococcal disease and receive annual influenza vaccination supported by Medicare reimbursement and institutional quality standards. But far fewer adults >18 years of age have received vaccines recommended by the ACIP based on age or risk, including influenza, pneumococcal, hepatitis A virus, hepatitis B virus, and herpes zoster vaccines [11].

The potential for vaccines to prevent disease and save lives in low-income countries was first addressed on a global scale in 1974 when the World Health Assembly introduced the Expanded Programme on Immunization (EPI) at a time when few children in these countries received any vaccine other than smallpox vaccine. Originally the EPI supported delivery of 4 additional vaccines: BCG, diphtheria–tetanus–whole cell pertussis vaccine (DTP), oral polio vaccine, and live attenuated measles vaccine [12]. The Universal Childhood Immunization initiative led by the United Nations Children’s Fund in collaboration with World Health Organization helped boost global coverage with 3 DTP doses to 80% by 1990. The establishment of Gavi, the Vaccine Alliance in 2000, a partnership of national governments and nongovernmental and philanthropic organizations, led to a sea change in immunization delivery to children, families, and communities in the world’s poorest nations by enhancing uptake of existing vaccines and by introducing newly available but more expensive vaccines into the EPI schedule, creating social equity exemplary of the most altruistic goals of global health. As a result, recent modeling predicts that immunization against 10 vaccine-preventable diseases will prevent 69 million deaths between 2000 and 2030 in 98 low- and middle-income countries [13], an estimate that is certainly low [14]. Accelerated vaccine development of new vaccines to reduce the burden of the prevalent global diseases including tuberculosis, human immunodeficiency virus, and malaria; improved regulatory review; continued strengthening of immunization delivery systems to enhance coverage; and introduction of novel vaccines for global health emergencies such as polio are key areas of focus that will improve the lives of persons living in the poorest regions of the world.

But vaccines do not save lives. Vaccinations save lives. Unfortunately, in the strange world we inhabit, we live with a paradox in which the benefits, as enormous as they are, are hidden from view by the absence of endemic disease, allowing some to distrust or outright reject vaccination. Although social and cultural contexts differ, immunization initiatives in high-, middle-, and low-income countries are similarly challenged by irrational beliefs rooted in shallow awareness, fear of adverse events, distrust of governmental authority, and suspicion of the motives of vaccine developers and manufacturers, which are enabled by the internet, the 24-hour news cycle, and self-styled “experts” who assert a litany of unsupportable theories and claims. While this supplement does not specifically address how to better understand and respond to those who hold and espouse antivaccination views, we must all be alert to the threat that waning confidence in immunization poses to our health and well-being.

Global distribution and use of effective and safe vaccines will be the shortest path to ending the coronavirus disease 2019 pandemic and reversing the medical and economic consequences. As this supplement goes to press, 75 candidate severe acute respiratory syndrome coronavirus 2 vaccines have entered clinical testing, 10 of these have received authorization for use by national regulatory authorities, and a global plan is being developed to assure vaccine access to the world’s poorest nations [15]. To equal the immense, unassailable impact of the highly effective vaccines that have come before, the new coronavirus vaccines will need to not only meet acceptable standards of safety and efficacy, but must also overcome obstacles bred of ignorance and mistrust to achieve sufficient levels of public acceptance to end the pandemic.

## Notes

***Supplement sponsorship.*** This supplement is sponsored by the Bill and Melinda Gates Foundation.

***Potential conflicts of interest.*** A. S. B. is employed by the Bill & Melinda Gates Foundation, and J. F. M. is a former employee of the Bill & Melinda Gates Foundation. All other authors report no potential conflicts of interest.
